# A Feature Extraction Method Based on Differential Entropy and Linear Discriminant Analysis for Emotion Recognition

**DOI:** 10.3390/s19071631

**Published:** 2019-04-05

**Authors:** Dong-Wei Chen, Rui Miao, Wei-Qi Yang, Yong Liang, Hao-Heng Chen, Lan Huang, Chun-Jian Deng, Na Han

**Affiliations:** 1 School of Electronic Information Engineering, University of Electronic Science and Technology of China, XueYuan Road, Shi Qi District, Zhongshan 528400, China; chendwzsc@zsc.edu.cn (D.-W.C.); yangwq18@gmail.com (W.-Q.Y.); greentree_2001@163.com (L.H.); dengcj80@126.com (C.-J.D.); 2 Faculty of Information Technology, Macau University of Science and Technology, Avenida Wai Long, Taipa, Macau 999078, China; miaorui.research@gmail.com (R.M.); yliang@must.edu.mo (Y.L.); chenhaoheng@foxmail.com (H.-H.C.); 3 School of Business, Beijing Institute of Technology, JinFeng Road, TangJiaWan Town, Zhuhai 519000, China

**Keywords:** emotion recognition, feature extraction, differential entropy, linear discriminant analysis, electroencephalography

## Abstract

Feature extraction of electroencephalography (EEG) signals plays a significant role in the wearable computing field. Due to the practical applications of EEG emotion calculation, researchers often use edge calculation to reduce data transmission times, however, as EEG involves a large amount of data, determining how to effectively extract features and reduce the amount of calculation is still the focus of abundant research. Researchers have proposed many EEG feature extraction methods. However, these methods have problems such as high time complexity and insufficient precision. The main purpose of this paper is to introduce an innovative method for obtaining reliable distinguishing features from EEG signals. This feature extraction method combines differential entropy with Linear Discriminant Analysis (LDA) that can be applied in feature extraction of emotional EEG signals. We use a three-category sentiment EEG dataset to conduct experiments. The experimental results show that the proposed feature extraction method can significantly improve the performance of the EEG classification: Compared with the result of the original dataset, the average accuracy increases by 68%, which is 7% higher than the result obtained when only using differential entropy in feature extraction. The total execution time shows that the proposed method has a lower time complexity.

## 1. Introduction

Electroencephalography (EEG) is a means of data acquisition via sensors [1,2]. In actual applications, due to the large amount of data involved in EEG, the collected data is often used in the mobile edge computing server for moving edge calculation. The Brain–Computer Interface (BCI), also known as the direct neural interface, is an interdisciplinary cutting-edge technology. It is a direct connection pathway established between a human or animal brain (or culture of brain cells) and an external device [3,4,5,6,7]. The role of BCI is to establish communication between the human brain and external computers or other intelligent electronic devices [8,9]. Emotional recognition is a very important part of BCI. Emotional recognition generally refers to the acquisition of an individual’s physiological or non-physiological signals to automatically identify the individual’s emotional state [10,11]. Emotional recognition is an important part of the emotional calculation, and has a very important meaning in medicine and engineering [12].

Feature extraction is a crucial step in accurately classifying or decoding EEG signals in a BCI system. Therefore, since the concept of BCI was proposed, researchers have conducted numerous studies on how to effectively extract features from EEG signals [13,14]. Since the original EEG signals are noisy, it is often difficult to find effective features directly by classification calculation. How to perform effective signal decomposition is an important problem. In response to this situation, early researchers mostly used statistical indicators (median, standard deviation, kurtosis symmetry, etc.) of the first difference of the signal for feature extraction, and then proposed the spectral density (EEG signal with a specific frequency band) [15], logarithmic power (Log BP) (band power based on the oscillation process in the signal) [16], Hjorth parameters (EEG signals described by activity, mobility, and complexity) [17], wavelet transform (decomposition of EEG signals) [15], etc. In 2013, Duan proposed a feature extraction method based on differential entropy, which achieved good results on emotion-based datasets [18]. In the past two years, many researchers have used differential entropy as a feature extraction method to achieve good results on different datasets [19,20]. However, researchers often use multi-channel acquisition equipment when collecting EEG data. This results in high-dimension single samples, which makes the model likely to be overfitted, and the high dimensionality will result in higher computational cost and time complexity of the model. Therefore, the feature extraction method that uses only differential entropy may cause the training of the model to be time-consuming and over-fitting for the multi-channel dataset.

In this situation, the dimension reduction used to perform quadratic feature extraction is an effective method. In previous studies, researchers have also experimented with many methods for feature extraction of EEG signals. For example, Lee proposed a data dimension reduction method based on principal component analysis (PCA), which significantly improved the classification results of the imaginary dataset of left- or-right-hand motor imagery data [21]. Subasi proposed a data reduction method based on linear discriminant analysis (LDA), which proved that the LDA method can effectively improve the classification of epileptic datasets [22]. These methods have been experimentally proven to effectively extract important features and reduce the dimensions of the sample. In this situation, we hope to combine signal decomposition and data dimensionality reduction in order to improve the accuracy of classification while reducing the computational time complexity.

**Figure 1:**
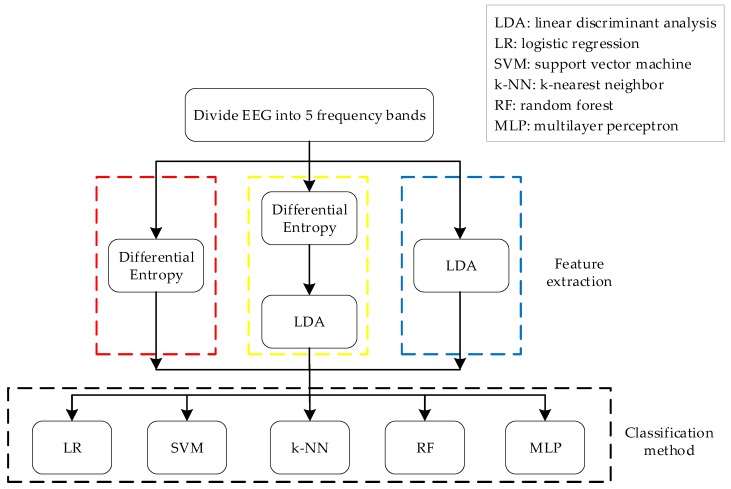
Flow chart of algorithm architecture.

This paper proposes a feature extraction method based on the fusion of differential entropy and LDA (Figure 1, yellow area). The method was tested on a three-category emotion EEG dataset. We performed experiments on the original dataset using differential entropy, LDA, and the fusion of differential entropy and LDA. We used five classic methods for classification: logistic regression (LR), support vector machine (SVM), k-nearest neighbor (k-NN), random forests (RF), and multilayer perceptron (MLP). At the same time, the cases using differential entropy alone (Figure 1, red area) and using LDA alone (Figure 1, blue area) as the feature extraction method are compared. The experimental results show that the proposed method can significantly improve the final classification results. The average accuracy is 68% higher than that of experiments on the original dataset and is 7% higher than the result when only using differential entropy for feature extraction. The execution time shows that the proposed method has less time complexity after feature selection. The method proposed in this paper has the following advantages compared with the previous methods: (i) compared with the method using only differential entropy and only the LDA method, the proposed method has higher classification accuracy; and (ii) the proposed method can effectively reduce the time complexity of the classification method. In general, this paper proposes a feature extraction method based on the fusion of differential entropy and LDA. The validity is verified based on the three-class emotion dataset, and the result is better than that of previous researches.

The remaining sections are organized as follows: Section 2 introduces the classification methods used in this paper, including LR, SVM, k-NN, RF, and MLP. Section 3 introduces the public dataset used in this paper and the fusion of differential entropy and the LDA method. Section 4 gives a performance comparison of experiments with different methods. In Section 5, the current state of the brain–computer interface and the emotional classification field are discussed. In Section 6, a conclusion is given and future work is described.

## 2. Related Works

This section introduces five classic machine-learning methods. In previous research, these methods have been applied to emotion recognition and other classification recognition based on EEG signals.

The logistic regression (LR) method is a classic classification method. This method is widely used in various fields including brainwave classification [23,24,25]. The LR method is used to solve the classification problem, firstly establish the cost function, and then iteratively solve the optimal model parameters through the optimization method. Finally, it verifies the quality of this model. In this paper, the L2 norm is used to prevent overfitting.

The SVM method is a classic machine-learning method. This method is widely used in the field of classification based on EEG signals [26,27,28]. If the data is not linearly separable, the kernel function will be used. Common kernel functions include linear, poly, radial basis function (RBF), sigmoid, and so on. In this paper, we use linear as a kernel function for the SVM method.

The k-NN method is a kind of classical data-mining method that has been used in EEG emotion recognition for many years [29,30]. The basis of the k-NN method is measuring the distance between different sample feature values. Its main concept is that for a new sample in the feature space, it belongs to the most frequent category in its most similar samples of k in all samples. In general, k is usually not greater than an integer of 20.

RF is an improved method of the decision-tree method. This method is also widely used in the classification of EEG signals [31,32,33]. RF is an algorithm that integrates multiple trees through the idea of integrated learning. Its basic unit is the decision tree, and its essence belongs to a large branch of machine-learning-integrated learning methods. From an intuitive point of view, each decision tree is a classifier, then for an input sample, N trees will have N classification results. The random forest integrates all the classification results by a voting strategy, so the category of highest frequency is the final output.

Finally, MLP, also called an artificial neural network, is a kind of neural network method which is often used in various fields, including EEG signal classification [34,35]. The MLP model consists of an input layer, an output layer, and multiple hidden layers. The layers are generally in the form of full connections, using the sigmoid or tanh functions as the activation function. MLP can implement nonlinear discriminants. Studies have shown that any function with continuous input and output can be approximated by MLP. An MLP neural network with a hidden layer (no limit to the number of hidden nodes) can learn any nonlinear function of the input to output.

## 3. Our Method

### 3.1. Dataset

The experiment was conducted using a public emotional EEG dataset called SEED, which uses film fragments as emotion-inducing materials and includes three categories: positive, neutral, and negative emotions. In each experiment, the participants watched movie clips of different emotional states. Each clip was played for about four minutes. In the experiment, three types of movie clips were played. Each type of movie clip contains five movies, giving a total of 15 movies. These movie clips were all from Chinese movies. There was a five-second prompt before each short film show, with 45 seconds of feedback time after playback and 15 seconds of rest after watching. A total of 15 subjects participated in the experiment (seven males, eight females, mean age 23.27 years old with a standard deviation of 2.37), all of whom had normal visual, auditory, and emotional states. The EEG signal when the subject was watching the movie was recorded through the electrode cap, with a sampling frequency of 1000 Hz. The experiment used the international 10-20 system and a 62-channel electrode cap. Each volunteer participated in three experiments, and each experiment was separated by about one week. Therefore, a total of 675 (15 × 15 × 3) data samples were formed. Then, 200 Hz down-sampling and 0.5–70 Hz filtering were performed to obtain a preprocessed EEG dataset. For more information on the dataset, please refer to the website http://bcmi.sjtu.edu.cn/~seed/index.html.

### 3.2. Methods

The method of using the differential entropy algorithm for feature extraction has been widely used in the field of image and signal processing [36,37]. This method can effectively extract information that may be valid in the sample. The feature extraction method proposed in this paper uses the differential entropy algorithm to decompose the signal, remove the noise of the EEG signal, and extract important features. LDA is a classic algorithm for pattern recognition. It was introduced in the field of pattern recognition and artificial intelligence by Belhumeur in 1997 [38]. The basic idea of LDA is to project high-dimensional samples into low-dimensional space to achieve the effect of extracting classification information and compressing feature space dimensions. After projection, the sample has the largest inter-class distance and the minimum intraclass distance. Therefore, it is an effective feature extraction method. In this paper, the LDA method is used to reduce the dimension of the data after signal decomposition, which is used to achieve the secondary extraction feature and reduce the time complexity of the classification method. This section describes a feature extraction method based on differential entropy and LDA. This section includes two parts: signal decomposition and data dimensionality reduction.

#### 3.2.1. Signal Decomposition

First, we perform signal decomposition on the original signal to remove noise and extract useful information from the signal. Entropy is a thermodynamic quantity describing the disorder of a system. The concept of entropy has been successfully applied to the analysis of EEG signals. Although the original EEG signal does not follow a fixed distribution, research has proven that from 2 to 44 Hz by steps of 2 Hz, the EEG signal after filtering obeys a Gaussian distribution. This paper uses differential entropy to perform signal decomposition of EEG [18].

Differential entropy is used to measure the complexity of continuous random variables, and is the entropy of continuous random variables. The differential entropy is also related to the minimum description length. Its calculation formula can be expressed as Equation (1):

(1) $$h(X)=-\int_{X}f(x)\log(f(x))dx$$

where *X* is a random variable and *f*(*x*) is the probability density function of *X*. For the time series *X* obeys Gaussian distribution *N* (*μ*, *σ*2), its differential entropy can be defined as Equation (2):

(2) $$h(X)=\frac{1}{2}\log(2\pi e\sigma^{2})$$

In a fixed frequency band *i*, the differential entropy is defined as Equation (3):

(3) $$h_{i}(X)=\frac{1}{2}\log(2\pi e\sigma_{i}^{2})$$

#### 3.2.2. Data Dimensionality Reduction

Next, we use the LDA method for quadratic feature extraction and data dimensionality reduction. There is a lot of noise in the EEG signals. However, the EEG signal after band-pass filtering has been proven to obey a Gaussian distribution [18]. More importantly, in a large amount of data, it can be sure that the EEG signal has a relatively obvious main component. This means that the EEG signals meet the requirements of the LDA method; that is to say, the signal conforms to a Gaussian distribution and has a distinct principal component. This in turn suggests that EEG signals can be extracted and classified using the LDA method. The goal of LDA is to create a new variable that is a combination of the original predictors. This is accomplished by maximizing the differences between the predefined groups, with respect to the new variable. The optimization function of the two-class classification and multi-class classification of the LDA method is different. Since this paper uses a three-category emotion EEG dataset, we give only a brief description of the method of multi-class classification.

Assumed dataset is $D=\{(x_{1},y_{1}),(x_{2},y_{2}),\ldots ,(x_{m},y_{m})\}$, any sample $x_{i}$ is n-dimensional vector, $y_{i}=\{C_{1},C_{2},\ldots ,C_{K}\}$. We define $N_{j}(j=1,2,\ldots ,K)$ as the number of samples of class j, $X_{j}(j=1,2,\ldots ,K)$ is a collection of class j samples, and $\mu_{j}(j=1,2,\ldots ,K)$ is the mean vector of the j sample. Define $\Sigma_{j}(j=1,2,\ldots ,K)$ as the covariance matrix for the class j samples. The $\mu_{j}$ is Equation (4), and the $\Sigma_{j}$ is Equation (5):

(4) $$\mu_{j}=\frac{1}{N_{j}}\sum_{x\in X_{j}}x$$

(5) $$\Sigma_{j}=\sum_{x\in X_{j}}\left(x-\mu_{j}\right)$$

Suppose the dimension of the low-dimensional space that the function needs to project into is *d*, the corresponding base vector is $\left(w_{1},w_{2},\ldots ,w_{d}\right)$, base vector composition matrix *W*. It is a matrix of n×d. The optimization objective function at this time is Equation (6). $S_{b}$ is Equation (7) and $S_{w}$ is Equation (8). $\prod_{\mathrm{diag}}A$ is the product of the main diagonal elements of A. The optimization process for J(W) can be converted to Equation (9):

(6) $$\underset{W}{\underset{︸}{\arg\max}}J(W)=\frac{\prod_{\mathrm{diag}}W^{T}S_{b}W}{\prod_{\mathrm{diag}}W^{T}S_{w}W}$$

(7) $$S_{b}=\sum_{j=1}^{K}N_{j}(\mu_{j}-\mu ){\left(\mu_{j}-\mu \right)}^{T}$$

(8) $$S_{w}=\sum_{j=1}^{K}S_{wj}=\sum_{j=1}^{K}{\sum_{x\in X_{j}}\left(x-\mu_{j})(x-\mu_{j}\right)}^{T}$$

(9) $$J(W)=\frac{\prod_{i=1}^{d}w_{i}^{T}S_{b}w_{i}}{\prod_{i=1}^{d}w_{i}^{T}S_{w}w_{i}}=\prod_{i=1}^{d}\frac{w_{i}^{T}S_{b}w_{i}}{w_{i}^{T}S_{w}w_{i}}$$

The process above is the feature extraction process.

## 4. Results

First we adopted a band filter to divide the EEG signals into five bands, including Delta (1–3 Hz), Theta (4–7 Hz), Alpha (8–13 Hz), Beta (14–30 Hz), and Gamma (31–50 Hz). Therefore, we obtained five datasets representing the corresponding band. Then, we combined the five-band data in order to inform the combined band data. After that, we applied differential entropy (DE), LDA, and the DE and LDA method proposed in this paper to extract features respectively. Finally, we adopted five classification methods, that is, k-NN, LR, MLP, RF, and SVM. Each test randomly assigned 675 samples into mutually exclusive training sets (70%) and validation sets (30%). The experiment adopted the five-fold cross-validation method to ensure the accuracy of the results.

For the five classifiers, parameter tuning was conducted. After tuning, the better parameters for these classifiers were chosen. For the logistic regression method, L2 penalty was used, and the inverse of regularization strength was 1.0. For the SVM method, the ‘rbf’ kernel function is applied, and the penalty parameter C was 1.0. For the k-NN method, the number of neighbors was set as 20. For the random forest model, the number of trees in the forest was 120, the maximum depth of the tree was 10, and the minimum number of samples required to split an internal node was 8. For the MLP classifier, the optimizer was the Adam method, the activation function was ReLU, and the batch size was 32; the size of two hidden layers were 64 and 32 respectively, and the learning rate was 0.0001.

We decided to adopt several evaluation methods to ensure the accuracy of the results. First, we calculated the accuracy to evaluate the method; the accuracy is defined as the ratio of the number of samples correctly classified by the classifier to the total number of samples in the test dataset. However, accuracy is not always valid for evaluating the performance of a method, especially in the situation that the numbers of true and false samples in the same datasets is not completely equal. Then, we calculated the F1 score, which is a statistical indicator used to measure the accuracy of a binary model. It also considers the accuracy and recall rate of the classification model. Moreover, we used the Kappa coefficient, which is generally thought to be a more robust measure than simple percentage agreement calculation. Finally, in order to judge the performance of the methods intuitively, we drew the confusion matrix graph and the box plot.

### 4.1. Classification Result of the Experiment with Different Feature Selection Methods

Table 1 gives the experimental outputs of prediction performance in the original dataset. Table 2 gives the experimental outputs of prediction performance in the original data based on LDA feature extraction. Table 3 gives the experimental outputs of prediction performance in the differential entropy data. Table 4 gives the experimental outputs of prediction performance in differential entropy data based on LDA feature extraction. These tables only show the best two classification methods in the experiments. Details are given in Supplementary Tables S1–S4.

Table 1 shows that the random forest method has the highest average accuracy in the Gamma band. Table 2 shows that the random forest method has the highest average accuracy in the case of combined frequency band data. Table 3 shows that the SVM method has the highest average accuracy in the case of combined frequency band data. Table 4 shows that the SVM method has the highest average accuracy in the case of combined frequency bands. It can be seen from Table 1, Table 2, Table 3 and Table 4 that, under different conditions, except for Table 1, in general, the accuracy of experiments on the combined frequency band data is better than the accuracy of experiments on the sub-band data, and the performance of the SVM method is superior to other methods. Therefore, the analysis of the experimental part uniformly uses the combined frequency band data and SVM method.

Table 1, Table 2, Table 3 and Table 4 show that, in the case of using the combined frequency band data, the classification effect of the differential entropy combined with LDA method is significantly better than the case of using the differential entropy method alone and the LDA method alone. The average classification accuracy of feature extraction using the differential entropy combined with LDA method is 82.5%, and the accuracy is 7.1% higher than that of the differential entropy method alone, 73.7% higher than that of the LDA method. The precision and the recall rate were improved by 4.3% and 4.2%, respectively, compared with the differential entropy method alone, and improved by 62.8% and 67.8%, respectively, compared with the LDA method alone. Compared with the feature extraction method of differential entropy alone and the LDA method alone, the F1 score increased by 4.2% and 69.3%, respectively. It is worth noting that the Kappa value of the differential entropy combined with LDA method is 0.698, which indicates that the predictive category of emotional EEG is highly consistent with the actual category, which is 59.5% higher than that of the LDA method alone and 6.7% higher than that of the differential entropy method alone. The experimental results show that the differential entropy combined with LDA method has better recognition effect than the existing methods based on the differential entropy method and LDA method.

The experimental results show that the differential entropy combined with LDA method can be effectively used to process EEG signals. The differential entropy method can extract the time–phase information of emotional EEG and reflect the change of emotion. The use of the LDA method preserves valid feature information while reducing the data dimension.

From the experimental results in the frequency bands of Delta, Theta, Alpha, Beta, Gamma, and the combined frequency bands data, it is found that the differential entropy combined with LDA method has the best classification effect, not only in the combined frequency band, but also in other frequency bands, and the classification result of the method proposed in this paper is also better. For example, in the Beta band, when using the SVM classification method, the average accuracy of the differential entropy combined with LDA method is 71.4%, which is 2.9% and 58.3% higher than the differential entropy method alone and the LDA method alone, respectively. The precision rate of the differential entropy combined with LDA method increased by 2.5% and 55.7% compared with the differential entropy method alone and the LDA method alone, respectively. The recall rate was increased by 2.4% and 63.8% compared with the differential entropy method alone and the LDA method alone, respectively. The F1 score was increased by 2.2% and 64.3% compared with the differential entropy method alone and the LDA method alone. The Kappa coefficient increased by 4.6% and 56.4%, respectively, compared to the differential entropy method alone and the LDA method alone. In the Gamma band, the average accuracy of the differential entropy combined with LDA method is 74.1%, which is 8.8% and 56.0% higher than the differential entropy method alone and the LDA method alone, respectively. The results show that the differential entropy combined with LDA method is superior to the existing differential entropy method and LDA method in the classification of emotional EEG in all frequency bands.

In order to analyze the prediction results more intuitively, this paper presents box plots of the accuracy rate of experiments in the combined frequency band, and a confusion matrix diagram for the prediction results of the best performing methods in each experiment.

**Figure 2:**
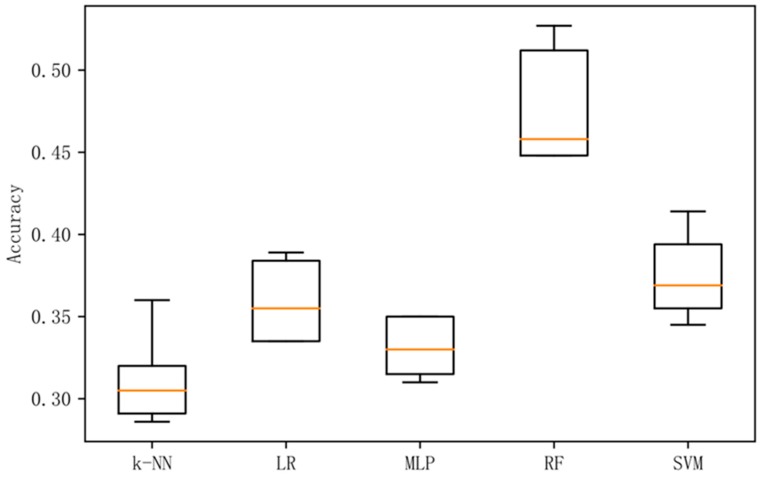
Accuracy in original dataset with five methods.

**Figure 3:**
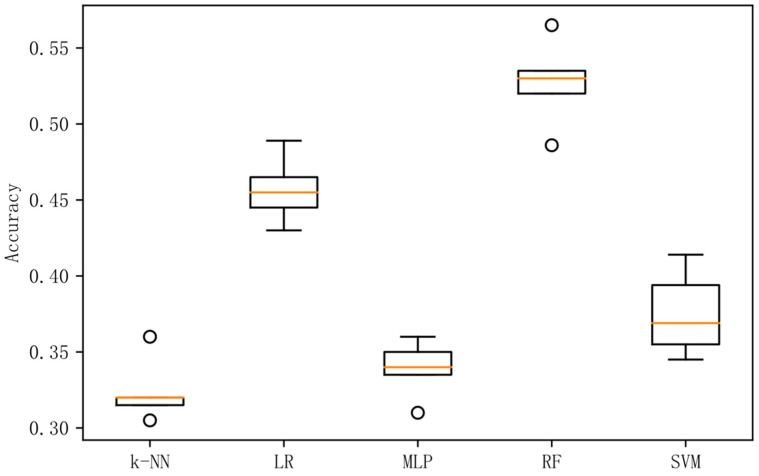
Accuracy in original dataset based on LDA with five methods.

**Figure 4:**
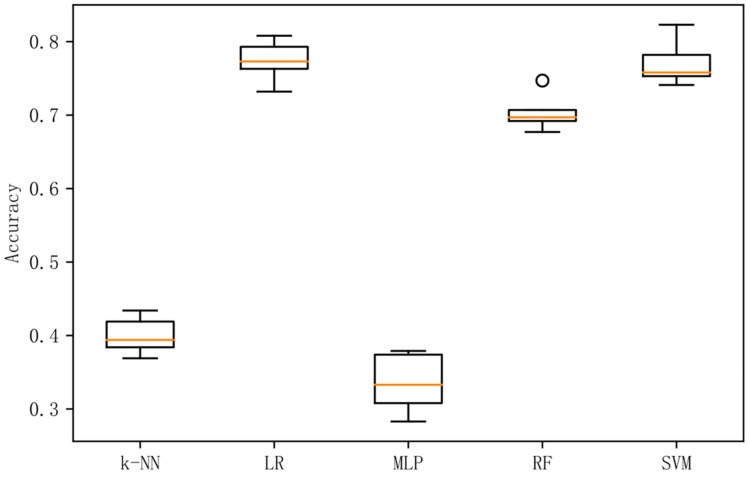
Accuracy in differential entropy dataset with five methods.

**Figure 5:**
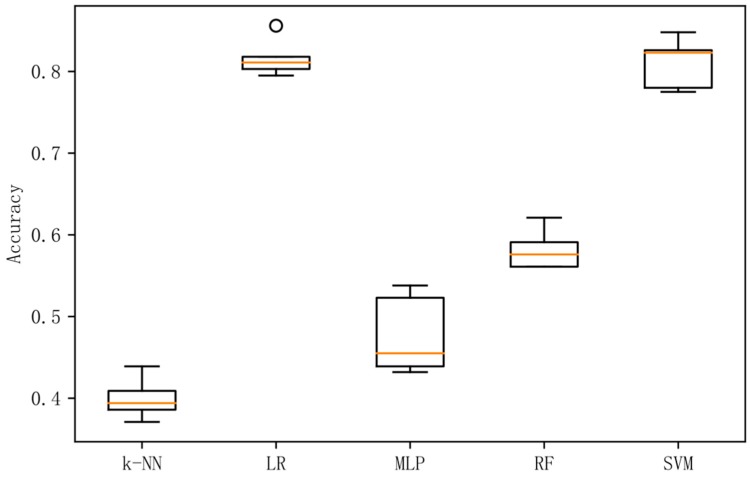
Accuracy in differential entropy dataset based on LDA with five methods.

Figure 2 shows a box plot of the classification accuracy for different classification methods on the original data. Figure 3 shows a box plot of the classification accuracy for different classification methods on raw data based on the LDA method. Figure 4 shows a box plot of the classification accuracy for different classification methods on raw data based on differential entropy method. Figure 5 is a box plot of the classification accuracy for different classification methods on raw data based on differential entropy combined with LDA method.

**Figure 6:**
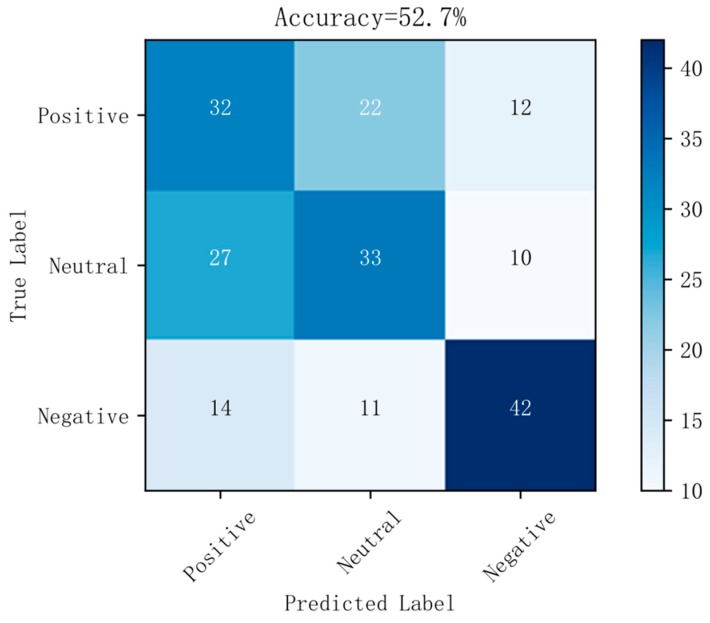
Confusion matrix in original dataset with RF.

**Figure 7:**
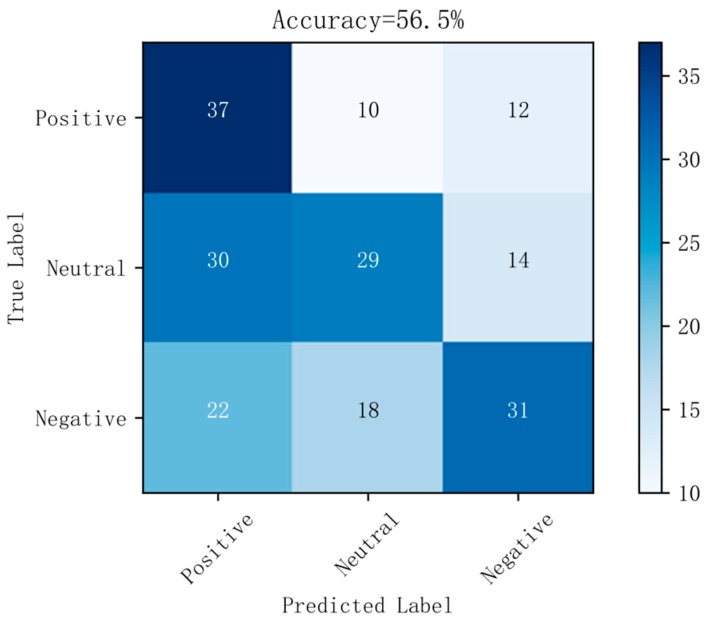
Confusion matrix in original dataset based on LDA with RF.

**Figure 8:**
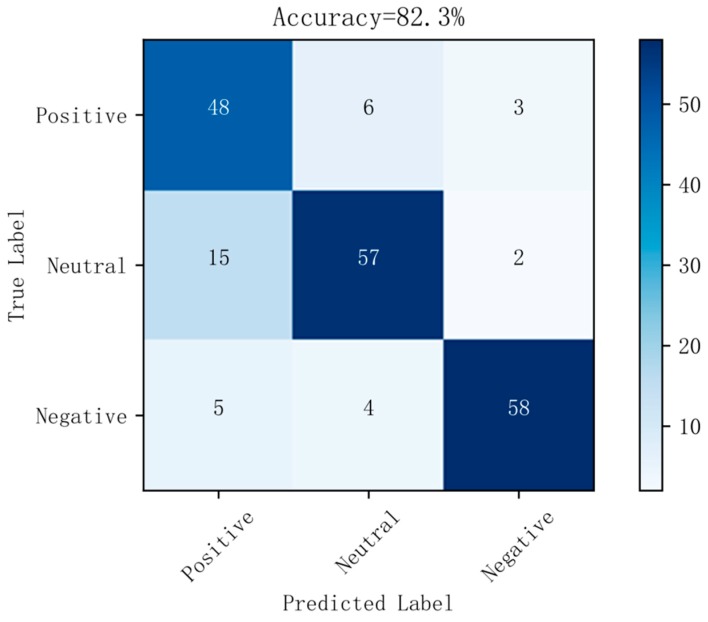
Confusion matrix in differential entropy dataset with LR.

**Figure 9:**
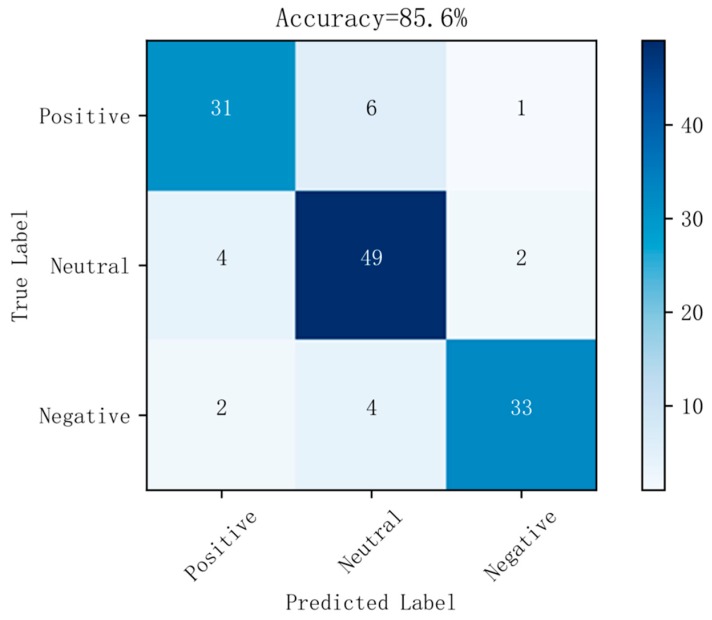
Confusion matrix in differential entropy dataset based on LDA with SVM.

Figure 6 shows a confusion matrix diagram of the best prediction results on raw data using the random forest method. Figure 7 shows a confusion matrix diagram of the best prediction results based on the LDA method using the random forest method. Figure 8 shows a confusion matrix diagram of the best prediction results based on the differential entropy method for feature extraction using the logistic regression method. Figure 9 shows a confusion matrix diagram of the best prediction result of feature extraction based on the differential entropy combined with LDA method using the SVM method.

It can be seen from Figure 2, Figure 3, Figure 4 and Figure 5 that the classification effect of the differential entropy combined with LDA method is superior to other methods. In the experiments based on the LDA method of raw data, the random forest method achieved the best accuracy. In the experiment based on the differential entropy method, the logistic regression method achieved the best classification accuracy, and the accuracy rate reached 77.4%. In the experiment based on differential entropy combined with LDA method, the SVM method achieved the best accuracy; the accuracy rate reached 82.5%, and the accuracy of the logistic regression method reached 81.7%. From the experimental results, the differential entropy combined with LDA method can effectively improve the results of emotional classification based on EEG.

It can also be seen from Figure 6, Figure 7, Figure 8 and Figure 9 that, in the confusion matrix diagram corresponding to the differential entropy combined with LDA method, the dark regions are concentrated on the diagonal, while the dark regions of other methods are relatively scattered. This indicates that the classification performance of differential entropy combined with LDA method for feature extraction of EEG signals is superior to other methods.

### 4.2. Time Complexity Result of Experiment with Different Feature Selection Methods

Table 5 shows the time complexity of four experiments with different classification methods in the original dataset, the original dataset based on LDA feature extraction, the differential entropy dataset, and the differential entropy dataset based on LDA feature extraction.

As can be seen from Table 5, the differential entropy combined with LDA feature extraction method has the best time complexity, and the time spent is significantly less than for other methods. Since the SVM method has the best classification effect in the combined frequency band under normal circumstances, the following is based on the experimental results of using the SVM method on the combined frequency band data. In experiments using the differential entropy method, the LDA method, and the differential entropy combined with LDA method, the time spent of the differential entropy combined with LDA method is 28.7% of that of the LDA method, and is only 17.6% of that of the differential entropy method. This indicates that the differential entropy method can extract the information of emotional changes of EEG signals, and that LDA can further extract effective features, accelerate the convergence speed of classification models, and reduce the time complexity. The experimental results show that the time performance of differential entropy combined with LDA method in emotional EEG recognition is better than the existing method.

## 5. Discussion

The last 10 years have seen the rapid development of computers and the continuous updating of signal acquisition equipment. New machine-learning and deep learning methods are constantly being proposed. The research on brain–computer interface has also made great progress, from the earliest two-category epilepsy recognition [39] and left-right hand movement imagination [40] to more complex two-classification motion recognition [41], and finally to multi-class emotion recognition [42]. We can see that the classification situation is increasing and the difficulty of feature extraction is increasing. This puts higher requirements on feature extraction methods and classification recognition methods. At present, with the depth of research, more complex feature-recognition and classification methods are constantly being proposed. Existing research has proved that the EEG signal is roughly consistent with a Gaussian distribution through band-pass filtering and signal processing, which lays a solid foundation for the feature extraction of EEG signals, such as signal feature extraction methods based on Fourier transformation, signal feature extraction methods based on differential entropy have been proposed. The proposed methods have greatly improved the classification results of EEG signals.

However, we believe that, whether in motion recognition or emotion recognition, it is necessary to have higher real-time performance in practical applications. In order to reduce the transmission delay, researchers often transmit the calculated data to a nearby mobile edge computing server for edge calculation instead of sending it to the cloud. However, due to the limited computing power of the server, the real-time transmission of EEG data is relatively high. In such a case, it becomes very important to perform effective feature extraction to reduce the amount of calculation. Under the premise of ensuring classification accuracy, the classification method needs to quickly judge the current state of the user. Therefore, it is not comprehensive enough to emphasize the improvement of classification accuracy. How to further reduce the time complexity of the method while improving the accuracy is still the focus of our attention. On this basis, the researchers have proposed dimensionality reduction methods such as PCA and LDA to reduce the time complexity of the classification method and to reduce the running time of the method.

Based on the previous research results and the characteristics of EEG signals, we believe that the combination of differential entropy and LDA method can effectively reduce the time complexity of the method while extracting EEG data features. The final experiment also verified this. Although our approach has many advantages, it is worth noting that our method can only reduce the complexity of the data in the feature extraction part; we cannot directly reduce the time complexity of the classification method. This has little effect on traditional machine learning methods. However, now, more and more researchers tend to use deep learning methods such as convolutional neural networks (CNN), recurrent neural networks (RNN), and deep belief network (DBN) [43,44,45]. Deep learning brings new breakthroughs in the use of brain–computer interfaces. Its classification accuracy is higher, and more complicated situations can be classified. However, due to the high dimension of EEG data and the relative scarcity of samples, determining how to establish a corresponding deep learning network is a big problem, and it is difficult to adjust the parameters of deep learning. Determining how to build a common model for different data is the direction of the next research. More importantly, these methods are inherently complex and can result in high time complexity for classification calculations. Therefore, our next goal is to optimize the deep learning method to further reduce the run-time requirements of the method, improve the classification accuracy, and try to build a more general deep learning model.

## 6. Conclusions

This paper proposes a feature extraction method based on the fusion of differential entropy and LDA method. Experiments were performed using five classical classification methods on emotion-based three-class datasets. The results show that the feature extraction method proposed in this paper can effectively improve the final classification accuracy in five classical classification methods. More importantly, the method proposed in this paper can reduce the time complexity and running time of the model. This means that the feature extraction method based on the fusion of differential entropy and the LDA method proposed in this paper can be effectively applied to multi-class emotion recognition. For the clinical environment, this means that if the method can be used, the patient’s mood and pathology can be judged more quickly, and doctors can better diagnose their condition and determine the patient’s state in real time. In the future, we hope to test more datasets and test the deep learning method.

## Figures and Tables

**Table 1:** Predictive performance of five methods using the original dataset.

Method	RF						SVM					
Band	Delta	Theta	Alpha	Beta	Gamma	Combined	Delta	Theta	Alpha	Beta	Gamma	Combined
Accuracy	0.385 ± 0.019	0.378 ± 0.029	0.365 ± 0.033	0.474 ± 0.030	0.491 ± 0.017	0.479 ± 0.038	0.310 ± 0.025	0.318 ± 0.028	0.330 ± 0.016	0.431 ± 0.021	0.315 ± 0.025	0.375 ± 0.028
Precision	0.383 ± 0.014	0.382 ± 0.030	0.378 ± 0.046	0.480 ± 0.039	0.487 ± 0.019	0.487 ± 0.032	0.311 ± 0.027	0.321 ± 0.028	0.333 ± 0.015	0.440 ± 0.026	0.324 ± 0.031	0.392 ± 0.024
Recall	0.391 ± 0.020	0.385 ± 0.031	0.373 ± 0.034	0.481 ± 0.030	0.496 ± 0.021	0.486 ± 0.035	0.311 ± 0.026	0.319 ± 0.027	0.333 ± 0.018	0.434 ± 0.022	0.320 ± 0.024	0.378 ± 0.031
F1 score	0.379 ± 0.016	0.373 ± 0.029	0.362 ± 0.035	0.465 ± 0.029	0.473 ± 0.012	0.474 ± 0.041	0.308 ± 0.024	0.315 ± 0.028	0.328 ± 0.015	0.429 ± 0.021	0.308 ± 0.025	0.372 ± 0.026
Kappa coef.	0.084 ± 0.028	0.074 ± 0.045	0.058 ± 0.052	0.219 ± 0.049	0.242 ± 0.029	0.227 ± 0.051	0.033 ± 0.038	0.020 ± 0.040	0.006 ± 0.025	0.322 ± 0.035	0.019 ± 0.036	0.069 ± 0.042

RF denotes the random forest method, and SVM denotes the support vector machine method. Kappa coef. is Cohen’s kappa coefficient. Each data field shows performance evaluation indices average ± std of 200 random splits of EEG samples.

**Table 2:** Predictive performance with five methods using the original dataset and Linear Discriminant Analysis (LDA).

Method	RF						SVM					
Band	Delta	Theta	Alpha	Beta	Gamma	Combined	Delta	Theta	Alpha	Beta	Gamma	Combined
Accuracy	0.342 ± 0.039	0.298 ± 0.043	0.322 ± 0.012	0.393 ± 0.035	0.414 ± 0.015	0.537 ± 0.028	0.310 ± 0.025	0.318 ± 0.028	0.330 ± 0.016	0.451 ± 0.020	0.475 ± 0.025	0.475 ± 0.028
Precision	0.353 ± 0.049	0.306 ± 0.050	0.329 ± 0.026	0.374 ± 0.044	0.381 ± 0.056	0.510 ± 0.040	0.311 ± 0.027	0.321 ± 0.028	0.333 ± 0.015	0.449 ± 0.026	0.464 ± 0.031	0.492 ± 0.024
Recall	0.350 ± 0.043	0.304 ± 0.044	0.335 ± 0.018	0.386 ± 0.028	0.394 ± 0.021	0.530 ± 0.028	0.311 ± 0.026	0.319 ± 0.027	0.333 ± 0.018	0.434 ± 0.022	0.466 ± 0.024	0.478 ± 0.031
F1 score	0.337 ± 0.036	0.290 ± 0.047	0.305 ± 0.015	0.385 ± 0.037	0.372 ± 0.023	0.523 ± 0.034	0.308 ± 0.024	0.315 ± 0.028	0.328 ± 0.015	0.429 ± 0.021	0.458 ± 0.025	0.472 ± 0.026
Kappa coef	0.024 ± 0.063	0.043 ± 0.063	0.002 ± 0.024	0.204 ± 0.041	0.227 ± 0.027	0.329 ± 0.039	0.033 ± 0.038	0.020 ± 0.040	0.006 ± 0.025	0.362 ± 0.035	0.319 ± 0.036	0.269 ± 0.042

RF denotes the random forest method, and SVM denotes the support vector machine method. Kappa coef. is Cohen’s kappa coefficient. Each data field shows performance evaluation indices average ± std of 200 random splits of EEG samples.

**Table 3:** Predictive performance with five methods using the differential entropy dataset.

Method	RF						SVM					
Band	Delta	Theta	Alpha	Beta	Gamma	Combined	Delta	Theta	Alpha	Beta	Gamma	Combined
Accuracy	0.525 ± 0.025	0.527 ± 0.053	0.513 ± 0.044	0.621 ± 0.044	0.627 ± 0.041	0.704 ± 0.026	0.568 ± 0.012	0.663 ± 0.034	0.601 ± 0.015	0.694 ± 0.036	0.681 ± 0.017	0.770 ± 0.030
Precision	0.530 ± 0.021	0.537 ± 0.055	0.529 ± 0.045	0.615 ± 0.046	0.620 ± 0.047	0.700 ± 0.029	0.568±0.014	0.667 ± 0.034	0.602 ± 0.015	0.692 ± 0.031	0.680 ± 0.025	0.768 ± 0.033
Recall	0.531 ± 0.028	0.532 ± 0.055	0.522 ± 0.039	0.624 ± 0.046	0.627 ± 0.047	0.704 ± 0.029	0.567 ± 0.013	0.666 ± 0.034	0.602 ± 0.016	0.694 ± 0.037	0.681 ± 0.024	0.770 ± 0.032
F1 score	0.525 ± 0.025	0.527 ± 0.053	0.509 ± 0.047	0.614 ± 0.046	0.617 ± 0.044	0.699 ± 0.029	0.565 ± 0.011	0.663 ± 0.035	0.599 ± 0.016	0.690 ± 0.038	0.677 ± 0.022	0.767 ± 0.032
Kappa coef	0.290 ± 0.036	0.294 ± 0.082	0.277 ± 0.063	0.433 ± 0.065	0.440 ± 0.063	0.556 ± 0.040	0.351 ± 0.018	0.496 ± 0.051	0.401 ± 0.023	0.541 ± 0.052	0.521 ± 0.027	0.654 ± 0.046

RF denotes the random forest method, and SVM denotes the support vector machine method. Kappa coef. is Cohen’s kappa coefficient. Each data field shows performance evaluation indices average ± std of 200 random splits of EEG samples.

**Table 4:** Predictive performance with five methods using the differential entropy dataset and LDA.

Method	RF						SVM					
Band	Delta	Theta	Alpha	Beta	Gamma	Combined	Delta	Theta	Alpha	Beta	Gamma	Combined
Accuracy	0.467 ± 0.017	0.484 ± 0.057	0.462 ± 0.049	0.556 ± 0.033	0.561 ± 0.035	0.582 ± 0.025	0.568 ± 0.012	0.663 ± 0.034	0.600 ± 0.015	0.714 ± 0.036	0.741 ± 0.017	0.825 ± 0.032
Precision	0.483 ± 0.020	0.517 ± 0.067	0.481 ± 0.048	0.570 ± 0.024	0.560 ± 0.035	0.590 ± 0.037	0.568 ± 0.014	0.667 ± 0.034	0.601 ± 0.016	0.709 ± 0.031	0.690 ± 0.025	0.801 ± 0.034
Recall	0.475 ± 0.015	0.496 ± 0.051	0.474 ± 0.047	0.568 ± 0.025	0.566 ± 0.031	0.593 ± 0.028	0.567 ± 0.013	0.666 ± 0.034	0.601 ± 0.017	0.711 ± 0.037	0.716 ± 0.024	0.802 ± 0.031
F1 score	0.453 ± 0.023	0.477 ± 0.065	0.454 ± 0.049	0.552 ± 0.033	0.555 ± 0.035	0.573 ± 0.031	0.565 ± 0.011	0.663 ± 0.035	0.598 ± 0.016	0.705 ± 0.038	0.677 ± 0.022	0.799 ± 0.033
Kappa coef	0.208 ± 0.023	0.238 ± 0.077	0.205 ± 0.071	0.343 ± 0.042	0.345 ± 0.051	0.378 ± 0.039	0.351 ± 0.018	0.496 ± 0.051	0.400 ± 0.023	0.566 ± 0.052	0.521 ± 0.027	0.698 ± 0.049

RF denotes the random forest method, and SVM denotes the support vector machine method. Kappa coef. is Cohen’s kappa coefficient. Each data field shows performance evaluation indices average ± std of 200 random splits of EEG samples.

**Table 5:** Complexity in four experiments with different methods.

Experiment	Method	Delta	Theta	Alpha	Beta	Gamma	Combined
Prediction performance in original dataset	kNN	5.215 ± 0.091	5.337 ± 0.349	6.077 ± 0.408	5.997 ± 0.347	6.205 ± 1.003	25.970 ± 0.069
	LR	111.323 ± 4.970	93.115 ± 7.211	97.788 ± 3.705	63.628 ± 4.623	51.596 ± 8.941	98.428 ± 6.571
	MLP	41.407 ± 14.262	50.474 ± 2.854	51.971 ± 1.282	50.837 ± 2.304	50.153 ± 2.195	105.568 ± 14.086
	RF	4.052 ± 0.115	4.497 ± 0.533	4.572 ± 0.280	4.982 ± 0.185	4.501 ± 0.386	9.544 ± 0.271
	SVM	19.429 ± 0.645	18.244 ± 1.475	19.388 ± 1.219	18.135 ± 0.993	17.517 ± 0.980	**79.412 ± 2.109**
Prediction performance in original dataset based on LDA	kNN	2.356 ± 0.043	2.310 ± 0.040	2.341 ± 0.068	2.329 ± 0.038	2.357 ± 0.030	15.060 ± 0.100
	LR	3.480 ± 0.142	3.162 ± 0.094	3.209 ± 0.078	3.320 ± 0.169	3.165 ± 0.078	16.151 ± 0.203
	MLP	2.726 ± 0.080	2.647 ± 0.052	2.680 ± 0.076	2.697 ± 0.054	2.686 ± 0.041	15.408 ± 0.127
	RF	2.845 ± 0.089	2.793 ± 0.062	2.836 ± 0.096	2.831 ± 0.065	2.815 ± 0.036	15.534 ± 0.153
	SVM	2.850 ± 0.075	2.539 ± 0.045	2.533 ± 0.076	2.477 ± 0.048	2.483 ± 0.024	**15.182 ± 0.097**
Prediction performance in differential entropy dataset	kNN	1.871 ± 0.007	1.876 ± 0.022	1.908 ± 0.011	1.889 ± 0.005	1.882 ± 0.012	9.370 ± 0.022
	LR	5.445 ± 0.310	7.340 ± 0.405	9.168 ± 0.986	10.321 ± 0.426	9.916 ± 0.558	26.932 ± 0.908
	MLP	6.524 ± 2.782	5.348 ± 1.719	4.159 ± 0.719	5.115 ± 1.640	4.865 ± 0.863	22.145 ± 3.354
	RF	2.243 ± 0.036	2.254 ± 0.019	2.269 ± 0.022	2.206 ± 0.018	2.192 ± 0.023	4.908 ± 0.140
	SVM	5.502 ± 0.019	5.266 ± 0.034	5.309 ± 0.059	4.125 ± 0.204	3.950 ± 0.213	**24.780 ± 0.163**
Prediction performance in differential entropy dataset based on LDA	kNN	0.838 ± 0.013	0.826 ± 0.027	0.823 ± 0.025	0.839 ± 0.011	0.841 ± 0.020	4.202 ± 0.079
	LR	1.001 ± 0.023	1.081 ± 0.032	1.134 ± 0.031	1.117 ± 0.018	1.097 ± 0.026	4.516 ± 0.100
	MLP	1.187 ± 0.023	1.162 ± 0.036	1.168 ± 0.038	1.187 ± 0.023	1.184 ± 0.015	4.668 ± 0.111
	RF	1.301 ± 0.019	1.302 ± 0.028	1.295 ± 0.027	1.309 ± 0.013	1.301 ± 0.029	4.819 ± 0.143
	SVM	0.966 ± 0.014	0.957 ± 0.029	0.973 ± 0.032	0.933 ± 0.022	0.928 ± 0.017	**4.366 ± 0.079**

LDA denotes the linear discriminant analysis method. kNN denotes the k-nearest neighbor method, and LR denotes the logistic method. RF denotes the random forest method, and SVM denotes the support vector machine method. Each data field shows the consumed time average ± std of 200 random splits of EEG samples.

## Supplementary Materials

The following are available online at https://www.mdpi.com/1424-8220/19/7/1631/s1.
